# Book Reviews

**Published:** 1889-02

**Authors:** 


### Book Reviews.

**A System of Gynecology by American Authors.** Edited by MATTHEW D. MANN, M. D., Professor of Obstetrics and Gynecology in the Medical Department of the University of Buffalo, N. Y. Vol. II. Pp. xiv., 1180; four colored plates; 361 wood engravings. Philadelphia: Lea Brothers & Co. 1888.

In reviewing a work like the present, there is more than an ordinary difficulty. Consisting, as it does, of detached chapters by various authors, there is a lack of compactness and unity, which the work and careful scrutiny of no editor, however faithful and able, can entirely overcome. If the volume were the product of one mind, its repetitions and inconsistencies could be pointed out without danger of appearing captious or hypercritical, while, as it stands, the general reader must himself recognize the points of variation, and reconcile them if he can; or, if he cannot, take what suits him, and account for the parallax of view by the theory of personal equation. So much that is found here has appeared elsewhere in a condensed form in individual works of gynecology, that the question forces itself upon us whether, in a work that is intended for the general practitioner, there is not such an expansion of the subject as will be tedious or perplexing. To the specialist there is so little new in the volume, that were it not for pre-advertisement of some of the authors' names, we believe there would be little temptation to purchase, excepting to prospect, as it were, the claims of some of the writers, whose names are not so well known in medical literature. We find in the index of authors, many familiar names whose writings we already have on our shelves, and greet them as old and tried friends, though they would fain surprise us in a new quarter, and in new company. Of these there is little necessity to speak.

The chapters on the Diseases of the Vagina and the Hysteroneuroses, respectively by Dr. C. C. Lee and Dr. G. T. Englemann, by which the volume is opened, are both well written, and the facts intelligently collated. In the first, the bibliography is abundant; and in the second, it is made evident that the uterus and its neuroses may be at the root of nearly every disease "that flesh is heir to." To the general practitioner, who has had his attention but little attracted to this phase of disease, the wide application of the term, together with the statement of cases, will be almost on the lines of romance. The chapter on Extra-uterine Gestation, coming, as it does, from Dr. T. G. Thomas, is worthy the most careful consideration. A full review of this chapter will, however, necessitate so much repetition in relation with a notice of Mr. Tait's monograph upon the same subject, that it is postponed until the formal notice of that book. The chapter upon Tumors of the Breast, by Professor Gross, has already received notice in these columns. The views of the author have already been embodied in an excellent monograph, and further comment is, therefore, uncalled for. Those ignorant of the book will do well to read it. Dr. Gross is a voracious reader, as well as a skilful collator of facts, and his experience along with these makes his opinion valuable. In the chapter following, upon Diseases of the Breast other than Tumors, by Dr. Park, the subject is intelligently considered. Chapters like this are of real value to the general practitioner. It is short, concise, and the methods of treatment are at once practical and rational. We are surprised, however, to find that the author, in the treatment of mammary abscess, has omitted to mention the beneficial results of strapping the mammæ. The omission is all the more striking, inasmuch as it perhaps offers the very best method of dealing with this trouble.

The Treatment of Fistulæ, by Dr. Edward W. Jenks, is one of the chapters overwhelming by the mass of materials presented. The bibliography is encyclopedic, and the discussion of the subject presents a variety that cannot fail to be perplexing to the general practitioner who goes to the book for information. We are led to believe that, in a work like the present, a simple statement of a simple method for dealing with the various fistulæ, would be more in harmony with the intelligent understanding of the general practitioner. The specialist or the teacher has already learned the various methods here presented. In passing, we cannot fail to notice that the operation accredited by Dr. Skene (Diseases of Women, 1888,) to Dr. Dudley, of Chicago, is here properly traced. We refer to suturing of fistula in ano, as in a recent tear.

The chapter on Diseases of the Bladder and Urethra, by Dr. W. H. Baker, is a full consideration of this important subject, covering about 100 pages. This chapter, like that by Dr. Park, is one of supreme importance not only to the gynecologist, but to every practitioner. Its consideration should be much fuller in every "Practice of Medicine." There is but little opportunity for the expression of new or startling facts or phenomena, but we find the subject intelligently considered, and indorse the therapeusis recommended as worthy the study and attention of all who meet it. The recommendation of the use of the benzoate of ammonia in vesical inflammation, is to be especially noticed. The remedy is often invaluable, and is just as often overlooked.

The study of the Non-malignant Tumors of the Uterus, by Dr. R. S. Sutton, is rather disappointing from a therapeutic standpoint. Any discussion of tumors of the fibroid type should, in view of the present electrical mania, take a stand either for or against, and, if possible, give a reason for any opinion held.

Mr. Keith's unpretending little book, while not pathological or speculative, is at once the type of matter clinical and instructive for one who will learn the peculiarities of such neoplasms. The bibliography of the chapter is full, though not recent.

The Malignant Diseases of the Uterus, by Dr. W. T. Lusk, considering its author and the importance of the subject, is for the gynecologist disappointing, because too short, and because the author has omitted almost altogether the bibliography of the affection. Apart from these shortcomings—and in a work like this we do not feel that they should be so considered—the discussion and enunciation of principles are wise, and in accordance with all that is best received at the present time.

Lacerations of the Cervix Uteri, by Dr. Bache McE. Emmet, next attracts attention. We think it unfortunate that he should have been called upon to rewrite a subject with which the name of the elder Emmet is so thoroughly associated. Dr. T. A. Emmet has so conspicuously made this subject his own, that any consideration of it from his standpoint not only seems like vain repetition, but like presumption on the part of the writer who attempts it. This, of course, puts such writer at once upon the defensive, and, therefore, at a disadvantage. The chapter is well written, and will prove instructive to those not already acquainted with the operation. We trust that ere long we shall hear from the author, in paths in which he will not be handicapped by the achievements of his illustrious kinsman and preceptor.

Chronic inversion of the uterus is a subject whose interest lies in the fact of its rarity. To the general practitioner it is an accident, for which, if it occur, consultation will at once commend itself. The specialist will find the subject briefly but sufficiently discussed.

Injuries and Lacerations of the Perineum and Pelvic Floor, by Dr. H. A. Kelly, is a lengthy and laboriously-illustrated presentation of the subject. The seemingly new consideration presented, as the prime feature of the article, is at once misleading and incorrectly vested with novelty, inasmuch as the operation of Dr. Emmet was specially directed to this condition under another name—laceration. The laceration in the so-called relaxation is just as real, as where a tear extends through the mucous membrane of the vagina to and through the mucous membrane of the rectum. It is of sufficient importance, in passing, to note one of the causes of injury from without. The writer says: "Several cases have come to my notice in which little girls have had their perineum torn from vagina to anus, opening the whole recto-vaginal septum, by sliding down a baluster, and striking the flat boss on the top of the newel-post." Dr. Kelly would do a real service to the gynecological profession if he would point out the literature of the cases to which he refers. Till such cases are substantiated, there are many who must look upon them as the mythology of possibilities. There is an unnecessary multiplication of drawings to illustrate the subject, and some of them are misleading and not to be followed. This is especially shown in Figs. 245 and 254. The buried point of the scissors cannot fail to do infinite harm if practised. The illustrations here are markedly inferior to those of Dr. Skene in his recent work on Gynecology. The chapter is remarkable more for the collection of various operations, than for anything new it proposes. Like operations on the cervix, operations upon the perineum usually begin with Emmet, and end somewhere within the lines by him proposed. We here find no exception to the rule.

We believe it had been wise had Dr. Emmet been asked to write his description, in both this chapter and that on lacerations of the cervix uteri, in a way suited for the understanding of the general practitioner. This would have saved us the disappointment of reading many pages of general directions for the operations proposed, only in the end to find that the Emmet operation is so far superior to all others, that no pains ought to be spared to master its peculiarities.

Dr. Goodell's paper is readable, and gives a fair résumé of the history and methods of dealing with ovarian and extra-ovarian tumors. He still holds to the antiseptic treatment in all its details except the spray, which he has abandoned in his private hospital. This, in view of his acknowledgment and appreciation of the fact that Keith and Tait—thus far the most successful ovariotomists—have wholly abandoned Listerism. Bantock's later results are also an argument for the abandonment of chemical antisepsis. When such operators have proven that chemicals need be no part of cleanliness, it appears that no further evidence is needed.

Lister, himself, acknowledges that abdominal surgery is not the camping-ground for his system. When results like these are possible without a technique, which in itself has in abdominal surgery a confessed danger, Bigelow's fears, quoted by Dr. Goodell, resolve themselves into fancies against which no protection is needed, save their shaking off. With the exception of the antiseptic (chemical) paraphernalia, we are in full harmony with Dr. Goodell's method of preparation for the abdominal section. Every operation should be considered a complex one, and preparations made for any possible emergency. In dealing with a diseased vermiform appendix, he believes that here, if anywhere, the cautery or special precaution is to be urged to prevent infection of the peritoneum. The caution as to losing sponges and instruments in the abdominal cavity, should be heeded by all. Of our own knowledge we are able to add three cases, none of which have been published, to Mr. Tait's list. Operators, as a rule, publish their successes, not their blunders. Dr. Goodell is conservative in his expression concerning the use of the drainage-tube, and may, we believe, be fairly set down as one who believes in its efficacy and is not ready to abandon it. He attributes hernia, in some instances, to the use of the tube. This we can believe, but that in all cases it predisposes to hernia, we cannot accept. Other causes are far in the ascendancy in the production of this trouble. The discussion of the accidents and complications of ovariotomy is worthy studied attention, especially of those who believe the procedure so simplified that it is within the abilities of all. The accidents and the complications are more a test of skill than the operation itself.

The next chapter is a discussion of the Diseases of the Ovaries, by Dr. Battey and Dr. Henry C. Coe. So far as the portion written by Dr. Battey is concerned, the space taken up with the question whether the priority of the procedure belongs to him or Mr. Tait, could have been better occupied. A book like that we are noticing is no place for medical polemics. Whether one or the other of these gentlemen proposed the operation, is of the utterest irrelevancy in a discussion of diseases of the ovaries. Dr. Battey, in describing the operation ("Battey's"), introduces a description of the vaginal method, and, at its close, says: "It is now doubtful whether any of these supposed advantages really exist in fact." The doubt is rather whether there is any excuse for applying the vaginal method at all.

Dr. Coe has no "hobby to ride," and accordingly has written an article deserving critical commendation. His discussion of the various forms of ovarian disease is clear and considerate. His study of prolapse of these organs can be received as being as thorough and reliable as anything upon the literature of the subject. Dr. Coe's labors extend still further into the succeeding chapter, which is written in conjunction with Dr. W. Gill Wylie. In discussing the neoplasms of the tubes, Dr. Coe retains the nomenclature commonly in vogue. This is wise. The terms exactly designate the condition found, and objections to their use are either from a desire to carp, or some other reason not very clear to those denying their correctness. Dr. Wylie has given us in the same chapter an excellent discussion of the diseases of the Fallopian tubes. As to the causation of salpingitis, we think he dismisses the gonorrheal theory a little too peremptorily. The question is not so much whether, for instance, in prostitutes there is other cause for tubal disease, as whether, in the absence of other cause than gonorrheal disease, this has not been present. The operative technique is well written and wisely stated. Dr. Wylie formulates his practical belief as follows: "In surgical practice I would place cleanliness first, drainage second, rest third, and antiseptics fourth." There will be some to dispute, but none to disprove, this belief. His advocacy of abdominal drenching or douching is also worthy of consideration. The bibliography of the chapter is full, but not exhaustive.

The pathology of Ovarian Tumors, by Dr. Stephen Y. Howell, is full and as satisfactory as can be hoped concerning a subject on which there is such a diversity of belief. Dr. Howell's chapter is of such nature that it would far better be in the shape of a neat monograph, where it would be much more likely to receive the credit it merits. The bibliography, while not exhaustive, is well selected, and will afford abundant resource for information to those desiring further discussion of the subject.

Of the article by the editor, Dr. Mann, an extended notice is not required. The clinical history and diagnosis of the various tumors are well stated, and constitute a readable portion of the book for the gynecologist.

The final chapter upon the Displacements of the Uterus, together with the considerations of prolapse of the vagina and uterus, is a brief and practical treatment of an important subject. The whole subject of displacements has been so thoroughly considered in another review in the preceding number, that to repeat here is unnecessary.

The book, though cumbersome, is well printed in clear type, and, like all publications of this house, is made up in handsome style.

---

**The Medical Bulletin Visiting List, or Physician's Call Record.** Arranged upon an original and convenient monthly and weekly plan, for the daily recording of professional visits. Philadelphia and London: F. A. Davis. 1889.

This new candidate for the favor of physicians possesses some unique and useful points. The necessity of rewriting names every week is obviated by a simple contrivance in the make-up of its pages, thus saving much valuable time, besides reducing the bulk of the book. It also contains the usual tables, formularies, calendars, and lists of antidotes for poisons, besides many other unusual features. Prices: No. 1, regular size, to accommodate seventy patients, weekly or monthly, $1.25; No. 2, large size, to accommodate 105 patients, weekly or monthly, $1.50. Address the publisher, 1231 Filbert street.

